# Optimizing Enzymatic Processes for Enhanced Nutritional and Organoleptic Properties of Chicken Bones

**DOI:** 10.3390/foods14071217

**Published:** 2025-03-31

**Authors:** Yuanyuan Zhang, Shengjiang Yu, Chang Liu, Shuai Jiang, Haili Wang, Yuliang Cheng, Yahui Guo, He Qian

**Affiliations:** 1 State Key Laboratory of Food Science and Resources, Jiangnan University, 1800 Lihu Road, Wuxi 214122, China; zyy10290222@163.com (Y.Z.); liuchang670250789@jiangnan.edu.cn (C.L.); highly@stu.jiangnan.edu.cn (H.W.); ylcheng@jiangnan.edu.cn (Y.C.); amtf168168@126.com (H.Q.); 2 School of Food Science and Technology, Jiangnan University, 1800 Lihu Road, Wuxi 214122, China; 3 Jichong Animal Nutrition Joint Research Center, Jiangnan University, Yichuang Industrial Park Building 5, Shaoxing 312000, China; bigjiang@jichongpf.com (S.Y.); shuai@jichongpf.com (S.J.); 4 School of Food and Bioengineering, Fujian Polytechnic Normal University, No. 1 Campus New Village, Longjiang Street, Fuqing, Fuzhou 350300, China

**Keywords:** enzymatic digestion, proteins, free amino acids, peptides, chicken by-products

## Abstract

The increasing demand for poultry products has led to significant by-products, with chicken bones being a rich source of proteins and minerals. The protease hydrolysis of chicken bones has emerged as a key method for extracting chicken bone protein. The objective of this study was to optimize enzyme combinations and hydrolysis reaction conditions to enhance both the nutritional value and quality of the product. Through univariate experiments and response surface methodology, the optimal enzymatic hydrolysis conditions were determined as follows: 55 °C, 1.5 h, and composite enzymes comprising papain (2.53%), bromelain (4%), and flavorzyme (4%) (*w*/*w*). The peptide content of the hydrolysis product obtained with the composite enzyme reached 336.78 mg/g, with small molecular peptides (<500 Da) accounting for 95% of the composite enzyme hydrolysis product. These small molecular peptides are more readily absorbed by the human body. Additionally, the free amino acids significantly increased, particularly those more easily absorbed by the human body such as glutamic, glycine, and aspartic. Moreover, there was a notable increase in the volatile flavor compounds including aldehydes and alcohols, which enhanced the flavor profile by producing fatty or mushroom-like aromas. This method enhances protein recovery and sample quality, converting chicken bone waste into valuable ingredients, contributing to sustainable food practices and innovative consumables for both pet and human consumption.

## 1. Introduction

Chicken meat production is a significant segment of the agricultural industry, with China’s chicken production expected to experience moderate growth during the 2024–2025 period [1,2]. The by-products of chicken processing are commonly utilized in the production of animal feed, plant fertilizers, flavors, and fragrances, albeit with relatively low added value [3,4]. Moreover, inadequate management of these by-products, particularly chicken bones, can result in serious environmental concerns and pose potential risks to human health [5]. In response to these issues, enzyme treatment has been explored as a promising approach to enhance the value of chicken bones and to investigate their potential biological activities, illustrating that biotransformation methods can effectively convert bones into a range of value-added products.

Chicken bones are abundant in proteins, minerals, and other essential nutrients, making them an effective source of nutritional supplements for supporting growth and skeletal development [6,7]. Enzymatic hydrolysis is the preferred technique for generating protein hydrolysates in the food industry due to its ability to modify the chemical, functional, and sensory characteristics of chicken bones [8,9]. Specifically, chicken bone hydrolysates can produce higher levels of free amino acids (FAAs), total nucleotide contents, and an enhanced flavor profile [10,11,12]. Alkalase has been shown to facilitate the formation of aroma precursors during the enzymatic digestion and heat treatment of sheep bones [13]. The enzymatic process breaks down proteins into polypeptides, which are further decomposed into dipeptides, tripeptides, and ultimately into individual amino acid molecules. Peptides derived from the hydrolysis process are more readily absorbed by the body [14]. These peptides have demonstrated considerable potential in managing various lifestyle-related diseases such as hypertension, hypercholesterolemia, and atherosclerosis. Their synthesis is influenced by factors such as raw material composition, seasonal variations, and the presence of endogenous enzymes [15]. The degree of hydrolysis can be precisely controlled to ensure the release of an optimal peptide content. Among the various methods, autolysis, which involves the use of endogenous enzymes, is a cost-effective approach, although it carries the risk of enzyme inactivation, potentially reducing the process’s controllability and reproducibility [16]. In contrast, exogenous enzymes offer a more reliable method, resulting in a soluble fraction that is rich in well-defined peptides and low in fat content. This fraction is ideal for enhancing flavor, functional ingredients, and nutritional additives in food products. The insoluble fraction, however, contains fat and other impurities, and can be repurposed for animal feed applications. Furthermore, the hydrolysis process releases amino acids that not only enhance the flavor profile of meat, but also serve as valuable nitrogen sources for sports nutrition and protein supplements, tailored to diverse dietary needs [17,18].

Numerous studies have investigated the enzymatic hydrolysis of chicken bones, focusing on the potential applications of the resulting hydrolysates [19,20,21]. Among these, the majority involve alkaline proteases, lipases, and pancreatic enzymes. However, there remains a gap in the literature concerning comparative studies that have examined enzyme-specific catalytic processes and their effects on product quality during enzymatic treatment. This study aimed to compare enzymatic hydrolysis using various commercial exogenous proteases and response surface-optimized composite enzymes, with the goal of producing short peptides and amino acids for industrial applications within a shorter timeframe. In the food industry, the enzymatic hydrolysis of chicken bones enables the extraction of protein hydrolysates rich in essential nutrients, which can be utilized as nutritional supplements to enhance the overall nutritional profile of food products. The peptides and amino acids derived from hydrolysis are not only readily absorbed by the human body, but also contribute to the enhancement in food flavor. The enzymatic process generates amino acids, peptides, and volatile flavor compounds (such as aldehydes, alcohols, and esters), all of which play a crucial role in improving the organoleptic properties of food. This process is particularly advantageous in meat processing, ready-to-eat meals, and pet food production, where it enhances both the taste and aroma. The enzymatic hydrolysis of chicken bones also provides a high-quality protein source, yielding nutrient-rich protein and amino acid additives for animal feed. These hydrolysates are especially valuable in pet food production (e.g., dog food, cat food), offering easily digestible nutrients that support the health and vitality of pets. Additionally, chicken bones, as by-products of the slaughtering process, can be converted into valuable resources through enzymatic hydrolysis, reducing waste and the environmental pollution associated with chicken bones, thereby aligning with sustainable development goals.

This study aimed to identify suitable enzymes and optimal enzymatic hydrolysis conditions for chicken bones by comparing various commercial exogenous proteases and their corresponding hydrolysis ranges. Response surface methodology (RSM) was used to optimize the enzyme combination, facilitating the production of short peptides and amino acids in a shorter time frame for industrial applications. The enhancement in the nutritional value of proteins, peptides, and free amino acids post-hydrolysis was evaluated by analyzing the concentrations of free amino acids, peptide levels, and molecular weight distribution. Additionally, the improvement in flavor was investigated through the analysis of volatile flavor compounds and flavor amino acids in the hydrolysis products. By optimizing the enzymatic hydrolysis process, this study sought to increase the peptide production efficiency, improve the overall quality of the hydrolysates, and reduce the production costs. Ultimately, this research contributes to the development of sustainable food systems by utilizing chicken bones via enzymatic hydrolysis to enhance their nutritional and functional properties, advancing the food industry’s efforts toward sustainability.

## 2. Materials and Methods

### 2.1. Materials

Chicken bones were obtained from Jichong Trading Co., Ltd. (Shaoxing, China); papain (43,000 u/g), bromelain (40,000 u/g) from the Shandong Azure Bio-Technology Co., Ltd. (Binzhou, China); leucine transaminase (18,000 u/g), neutral protease (200,000 u/g), alkaline protease (360,000 u/g), and flavorzyme (200,000 u/g) were from Wuxi Yupuke Biotechnology Co., Ltd. (Wuxi, China); and keratinase (200,000 u/g) was from Shandong Fengtai Biotechnology Co., Ltd. (Jinan, China). The specific information of the selected proteases is shown in Table A1.

### 2.2. Optimization of Enzymatic Conditions for Peptide Extraction from Chicken Processing Bones

Sample preparation: Chicken bones were thawed, minced, and homogenized prior to experimentation. Under optimal pH conditions for protease activity, 2.0% protease (based on enzyme mass fraction) was added for enzymatic digestion at 55 °C in a water bath. After 3 h of digestion, the reaction was terminated by transferring the mixture to a 90 °C water bath for enzyme inactivation, maintaining the temperature for 15 min. The hydrolysis was then stopped by rapid cooling, followed by centrifugation (5000× *g*, 20 min) to remove floating fat and insoluble residues. The resulting digest was subsequently used for further experiments.

One-way experimental design: Based on the above procedure, one-way experiments were conducted to optimize the key variables of enzyme mass fraction (E/S), digestion time, and digestion temperature. As shown in Table 1, each experiment was repeated three times. The primary evaluation criteria were the degree of hydrolysis (DH) and the mass concentration of short peptides. Additionally, the flavor and nutrient content of the hydrolysates were assessed to determine the most suitable enzymes for enzyme compounding and to establish the optimal enzymatic conditions.

Response surface experiment: The experimental design was based on a one-way design with the aim to identify the optimal enzymatic temperature conditions and enzyme concentrations to maximize peptide production. Specifically, the enzymatic digestion process revealed that 55 °C was the optimal temperature range, and enzyme addition levels of approximately 1.5–3% were ideal for different enzymes. Alkaline protease, papain, bromelain, and flavor protease exhibited superior performance in peptide production. To further optimize the enzyme combination ratio, the central composite design (CCD) method was employed to determine the optimal combination of variables for maximizing the short peptide yield (Table 2 and Table 3). The experimental design included twenty-four factorial points with five replicates at the center point, with each experiment being repeated three times.

### 2.3. Degree of Hydrolysis

The degree of hydrolysis (DH): The degree of hydrolysis (DH) was defined as the percentage of peptide bonds broken (h) relative to the total number of peptide bonds per unit weight (h_tot_), as determined using the pH-stat method [22]. To determine DH, 60 mL of distilled water was added to 5 mL of enzyme-digested chicken pulp, and the pH was adjusted to 8.20 using a precision pH meter. Then, 20 mL of formaldehyde was added, and the pH was titrated to 9.20 using 0.1000 M NaOH as the standard titrant. The volume (V_1_) of NaOH consumed was recorded. A blank experiment was conducted by replacing the sample with distilled water, and the volume (V_0_) of NaOH consumed in the blank was also recorded. The concentration of α-NH radicals (μmol/mL) and the degree of hydrolysis were calculated. The experiment was conducted in triplicate, and the average value was used in the final calculation, which was determined using the following equation:

(1) $$Hydrolysis\;degree(DH)/\%=\frac{h}{h_{tot}}\times 100$$

### 2.4. Determination of Peptide Mass Concentration

Peptide mass concentration determination: The mass concentration of peptides was determined using the biuret-trichloroacetic acid method [23,24]. Glutathione (GSH) was used as the standard, with concentrations ranging from 0 to 4 mg/mL. To prepare the standard solutions, 2 mL of GSH at varying concentrations was mixed thoroughly with 3 mL of disulfiram reagent and allowed to stand for 30 min. The absorbance of the standard samples was measured at 540 nm using an automated enzyme marker (Berten Instruments, Rockville, MD, USA), and a standard curve was constructed. For the analysis of enzyme-digested products, 4 mL of enzyme-digested chicken bone slurry was mixed with an equal volume of 10% trichloroacetic acid. The mixture was well-mixed and centrifuged at 4 °C and 4000 rpm for 20 min. The supernatant was then diluted by a factor of n, and 2 mL of the diluted supernatant was combined with 3 mL of biuret reagent. After standing for 30 min, the absorbance was measured at 540 nm. All experiments were performed in triplicate, and the average value was used for calculation. Finally, the peptide mass concentration in the digested slurry was calculated using the standard curve, according to the following equation, where A represents the absorbance and n is the dilution factor:

(2) $$Peptide\;mass\;concentration=\frac{A-0.1105}{0.0375}\times n$$

### 2.5. Determination of Free Amino Acids

We added 15 mL of 5% (*w*/*v*) trichloroacetic acid (TCA) to exactly 1.0 g of lyophilized enzymatically digested meat slurry powder, homogenized it for 1 min in an ice bath, transferred it to a 25 mL volumetric flask, extracted it with ultrasonication for 20 min, and then left it for more than 2 h. The sample was filtered through a double-layer filter paper and passed through a 0.22 μm aqueous membrane. The sample was placed into 400 μL liquid phase vials. The identification and quantification of free amino acids were performed by an Agilent HPLC system (Agilent Technologies, Agilent Technologies Inc., Santa Clara, CA, USA) equipped with an ODS-2 HypersilTM (5 µm, 4.6 × 250 mm). The mobile phase A consisted of a solvent blend containing 0.4 M NaAc, 0.02% (*v*/*v*) TEA, and 0.5% (*v*/*v*) THF at pH 7.2, whereas the mobile phase B comprised a solvent mixture of 0.4 M NaAc, 40% (*v*/*v*) ACN and 40% (*v*/*v*) MeOH in pH 7.2. The separation was performed at a flow rate of 1 mL/min. The injection volume was 10 μL. The column temperature and detection wavelength were maintained at 40 °C and 338 nm, respectively. The elution conditions were as follows: 0–27.5 min, 8% B; 27.5–31.5 min, 60–100% B; 31.5–35.5 min, 100% B; 35.5–40 min, 8% B. The free amino acid levels were calculated based on the calibration curve and expressed as mg/g dry weight of lyophilized enzymatically digested meat slurry powder.

### 2.6. Determination of Peptide Molecular Weight Distribution

The molecular weight distribution of the peptides was determined using HPLC (HPLC, Waters 2695, Waters Corporation, Milford, MA, USA), equipped with a 2487 ultraviolet detector and Empower Workstation GPC software (Waters Corporation, Milford, MA, USA). For sample preparation, 100 mg of the lyophilized enzymatically digested meat slurry powder was dissolved in water in a 10 mL volumetric flask, sonicated for 5 min, centrifuged at 10,000 rpm for 5 min, and then filtered through a 0.22 μm filter membrane prior to injection. The chromatographic conditions were as follows: a TSKgel2000SWXL column (300 mm × 7.8 mm), mobile phase consisting of acetonitrile/water/trifluoroacetic acid in a ratio of 40/60/0.1 (*v*/*v*/*v*), flow rate of 0.5 mL/min, column temperature maintained at 30 °C, and an injection volume of 10 μL.

### 2.7. Separation and Identification of Volatile Flavor Components of Enzymatic Digests by Headspace Solid-Phase Microextraction Gas-Phase High-Throughput Time-of-Flight Mass Spectrometry (HS-SPME-GC-TOF-MS)

The method described by Zihan Zhang [25] was applied with slight modifications for the analysis of volatile substances using GC–TOF–MS (Pegasus BT, Leco Corporation, St Joseph, MI, USA). A precise 4.0 g sample of lyophilized chicken pulp was weighed using an analytical balance and placed in an extraction flask containing 8 mL of saturated saline. To this, 10 μL of 2-methyl-3-heptanone at a concentration of 0.8 mg/mL was added. The flask was tightly sealed, and the mixture was shaken to ensure equilibration at 40 °C for 15 min. Subsequently, a DVB-CAR-PDMS solid-phase microextraction (SPME) fiber was inserted into the flask and maintained in a constant temperature water bath at 40 °C for 30 min. After extraction, the fiber was removed and immediately inserted into the GC injection port for thermal desorption at 260 °C for 2 min. Before injection, the fiber was conditioned in the inlet port at 270 °C with a helium flow for 1 h to eliminate any residuals between samples.

Volatile flavor components were resolved by the GC-TOF-MS technique under the following conditions:

GC conditions: HP-5MS column, 30 m × 0.25 mm; helium flow rate: 1.5 mL/min; non-split injection; inlet temperature: 250 °C. The heating program was as follows: held at 35 °C for 3 min, then ramped at 3 °C/min to 70 °C, followed by a 10 °C/min increase to 200 °C, and finally a 20 °C/min ramp to 260 °C, held for 5 min.

MS conditions: Ion source: 70 eV; ion temperature: 230 °C; interface temperature: 250 °C; mass range: 30–400 amu.

Qualitative analysis: The MS spectra were compared with the National Institute of Standards and Technology (NIST) database to identify the compounds. The match score for the identified volatile flavors was greater than 80, with the highest match reaching 100.

Quantitative analysis: The internal standard method was employed to calculate the content of the flavor compounds by comparing the concentration and peak area of the internal standard, 2-methyl-3-heptanone, to that of the target compounds.

### 2.8. Statistical Analysis

Statistical analyses were performed using Origin 2017 for one-way analysis and peptide molecular weight distribution. Response surface experiments were designed and analyzed using Design-Expert 10 software. The free amino acids were first subjected to statistical analysis using GraphPad Prism (version 8.0) software, where the significance of *p*-values (*p* < 0.05) was determined using one-way analysis of variance (ANOVA) and Tukey’s multiple comparisons test. For volatile flavor substances, a one-way analysis of variance (*t*-test) was used to calculate the statistical significance (*p*-value) and fold change (FC) of the metabolites between the two groups. Differential metabolites were defined as those with a VIP value > 1, *p*-value < 0.05, and FC ≥ 2 or FC ≤ 0.5.

## 3. Results

### 3.1. One-Way Experimental Design of Seven Enzymes

Based on the action sites of different proteases reported in the literature, seven endonucleases and exonucleases were selected for the enzymatic hydrolysis of chicken bones in this study. These enzymes were chosen for their specific cleavage sites and enzymatic properties, which are crucial in determining the hydrolysis efficiency and peptide composition.

**Figure 1:**
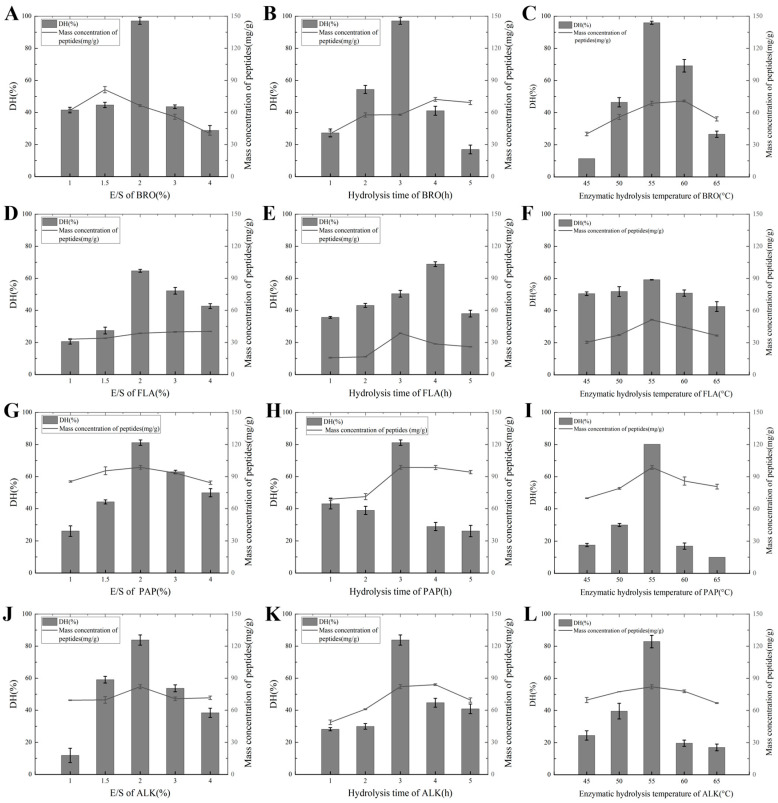
(**A**) DH and peptide mass concentration under the influence of the E/S of BRO; (**B**) DH and peptide mass concentration under the influence of the hydrolysis time of BRO; (**C**) DH and peptide mass concentration under the influence of the hydrolysis temperature of BRO; (**D**) DH and peptide mass concentration under the influence of the E/S of FLA; (**E**) DH and peptide mass concentration under the influence of the hydrolysis time of FLA; (**F**) DH and peptide mass concentration under the influence of the hydrolysis temperature of FLA; (**G**) DH and peptide mass concentration under the influence of the E/S of PAP; (**H**) DH and peptide mass concentration under the influence of the hydrolysis time of PAP; (**I**) DH and peptide mass concentration under the influence of the hydrolysis temperature of PAP; (**J**) DH and peptide mass concentration under the influence of the E/S of ALK; (**K**) DH and peptide mass concentration under the influence of the hydrolysis time of ALK; (**L**) DH and peptide mass concentration under the influence of the hydrolysis temperature of ALK.

**Figure A1:**
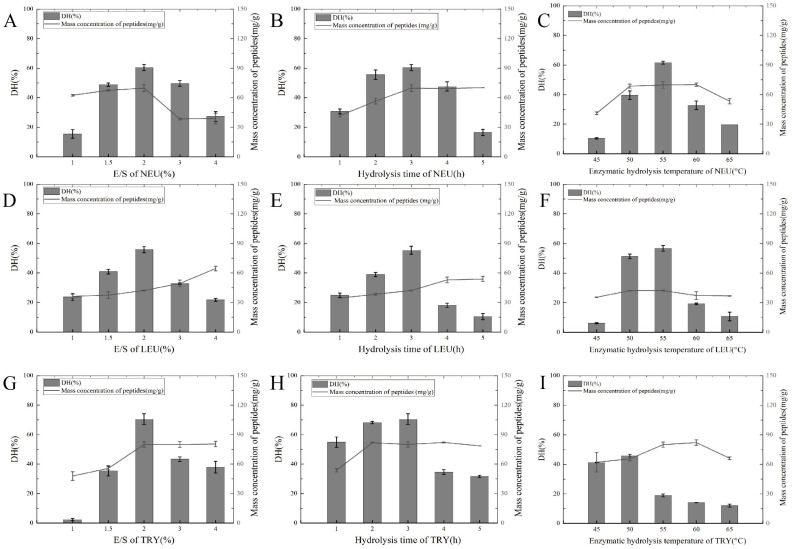
(**A**) DH and peptide mass concentration under the influence of the E/S of NEU; (**B**) DH and peptide mass concentration under the influence of the hydrolysis time of NEU; (**C**) DH and peptide mass concentration under the influence of the hydrolysis temperature of NEU; (**D**) DH and peptide mass concentration under the influence of the E/S of LEU; (**E**) DH and peptide mass concentration under the influence of the hydrolysis time of LEU; (**F**) DH and peptide mass concentration under the influence of the hydrolysis temperature of LEU; (**G**) DH and peptide mass concentration under the influence of the E/S of TRY; (**H**) DH and peptide mass concentration under the influence of the hydrolysis time of TRY; (**I**) DH and peptide mass concentration under the influence of the hydrolysis temperature of TRY.

The degree of hydrolysis (DH) is a key parameter that reflects the extent of the enzymatic cleavage of protein molecules and is directly correlated with the peptide bond cleavage in protein hydrolysates. As indicated in Table A1, the DH can vary significantly depending on the enzyme type and its source. Different proteases exhibit unique specificity for peptide bond cleavage due to their structural characteristics and active site configurations [26]. For example, non-specific endonucleases such as alkaline protease (ALK) hydrolyze carboxyl groups with aromatic or hydrophobic amino acids. Bromelain (BRO), which targets protein substrates and smaller peptides, works synergistically with ALK by complementing its enzymatic activity and broadening the spectrum of hydrolysis. Papain (PAP), on the other hand, exhibits a higher specificity for cleaving peptide bonds associated with alkaline amino acids, leucine, or glycine residues, making it effective in breaking down more rigid peptide structures. In contrast, trypsin (TRY) and neutral protease (NEU) have narrower hydrolytic ranges compared with ALK, PAP, and bromelain, resulting in lower water solubility rates and reduced peptide production (see Figure A1A–C,G–I and Figure 1A–C,G–L). In addition, flavorzyme (FLA), which contains both aminopeptidases and carboxypeptidases, plays an important role in selectively cleaving peptide bonds at the terminal positions of peptides, enhancing the release of free amino acids. Meanwhile, leucine transaminase (LEU) operates as an exonuclease, primarily acting on the terminal amino acids of peptide chains.

During the enzymatic hydrolysis process, adjusting the pH can introduce additional salts, which are difficult to remove in subsequent steps. Since the natural pH of the chicken slurry falls within the optimal pH range for these enzymes, the pH of the enzymatic hydrolysis reaction was not adjusted. When optimizing the enzymatic hydrolysis temperature, the enzyme-catalyzed reaction rate increases with the rise in temperature. However, once the temperature reaches a certain point, the reaction rate rapidly decreases due to the thermal denaturation of the enzyme protein. The temperature at which the enzyme-catalyzed reaction rate reaches its maximum value is referred to as the optimal temperature of the enzyme. When optimizing the enzyme amount, at lower substrate concentrations, the reaction rate increases with the increase in substrate concentration. However, when the substrate concentration in the reaction system is sufficiently high, substrate competition occurs, thereby inhibiting further reaction progress [27]. Regarding the optimization of enzymatic hydrolysis time, time is calculated starting from the moment the sample is placed into the water bath that has not yet been heated. As the water bath gradually heats up, the maximum peptide concentration is typically achieved between the 2nd and 3rd hour after reaching the optimal temperature. With the passage of time, the enzyme gradually loses its activity, and the rate of hydrolysis decreases. Even if the hydrolysis time is extended, the produced hydrolysate and the degraded protein may rebind, forming original proteins and resulting in a reduction in the final yield of peptides and amino acids [27].

The peptide mass concentration of the PAP digest, which exhibited the highest degree of hydrolysis (DH), was 125.01 mg/g, while the BRO digest demonstrated an even higher peptide mass concentration of 127.76 mg/g. Within the optimal enzymatic hydrolysis conditions, the peptide mass concentrations observed for ALK, PAP, BRO, and FLA were significantly higher than those of other proteases, which aligned with the results of the hydrolysis degree analysis. This correlation suggests that the degree of hydrolysis plays a key role in influencing the peptide mass concentration. Specifically, it is likely that the specific cleavage sites targeted by each enzyme contribute to variations in the hydrolysis efficiency and peptide yield. Enzymes such as PAP and BRO, which exhibit higher cleavage activity and broader specificity, may promote the formation of a greater concentration of peptides, thus achieving higher yields. On the other hand, proteases with more limited activity, or those with a narrower range of targeted cleavage sites, may result in lower peptide concentrations. Therefore, it can be concluded that both the enzyme type and the degree of hydrolysis directly affect the final peptide concentration, which in turn impacts the potential for further applications in various industries such as nutrition and food processing [28].

### 3.2. Response Surface Optimization Tests for Composite Enzymes

**Figure 2:**
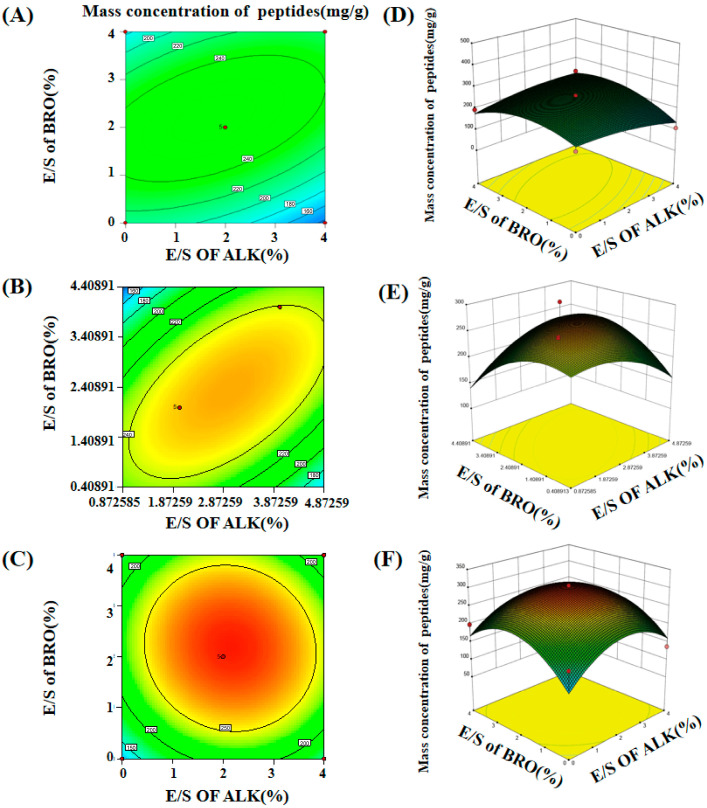
The effect of different complex enzymes on the peptide mass concentration at 1.5 h, 3 h, and 4.5 h respectively. (**A**–**C**) Contour plots of 1.5 h, 3 h, and 4.5 h; (**D**–**F**) response surface plots of 1.5 h, 3 h, and 4.5 h.

Based on the results obtained from the one-way experimental design, optimal conditions were established for the subsequent experiments. The optimal enzyme digestion temperature was determined to be 55 °C, as it was found to be the most conducive to the hydrolysis process. The enzyme mass fraction and reaction time were identified as the key factors influencing the enzymatic digestion process, with the enzyme concentration and time having the most significant impact on the hydrolysis efficiency. To further optimize peptide production, four enzymes—papain (PAP), bromelain (BRO), flavorzyme (FLA), and alkaline protease (ALK)—which are known for their high hydrolysis rates and ability to produce short peptides, were selected for enzyme compounding. The concentrations of these enzymes were varied between 0% and 4%, and three different hydrolysis durations—1.5 h, 3 h, and 4.5 h—were tested. These three time intervals were specifically chosen to assess the optimal enzymatic conditions for each protease. The optimization of these experimental parameters was based on the observed effects of time on the peptide yield and hydrolysis efficiency (Figure 2). Furthermore, the interactions between different enzyme ratios and their influence on the peptide mass concentration during each of the three time intervals were also investigated and optimized to determine the most efficient enzyme combinations for enhanced peptide production.

The optimization results obtained using the response surface methodology were derived from quadratic regression equations for three different hydrolysis durations at 55 °C. The fitted values of these quadratic regression equations were as follows: 646.79 (*p* < 0.05) for 1.5 h, 215.92 (*p* < 0.05) for 3 h, and 125.31 (*p* < 0.05) for 4.5 h. All results were statistically significant, demonstrating that the quadratic regression model provided a good fit for the experimental data. The low coefficient of variation (CV), ranging from 0.58% to 0.62%, further highlighted the minimal experimental error, indicating high precision in the measurement and reproducibility of the experiments. The correlation coefficients (R^2^) for the three different durations were 0.72, 0.77, and 0.83, respectively, which suggests that the quadratic regression model is highly suitable for analyzing and optimizing the enzymatic processes in composite meat slurry. These findings confirm the reliability and robustness of the model in predicting the behavior of enzymatic hydrolysis and its corresponding effects on peptide production.

Y = 256.71 − 7.13 × A + 13.4 × B + 46.53 × C − 14.5 × D + 30.06 × AB − 44.67 × AC − 32.16 × AD − 9.06 × BC + 53.75 × BD + 55.08 × CD − 18.46 × A^2^ − 52.66 × B^2^ − 35.55 × C^2^ − 30.04 × D^2^ (3)

The optimal parameters for enzymatic digestion extraction were identified as follows: a temperature of 55 °C, a reaction time of 1.5 h, and an enzyme mass fraction of 2.53%. Under these conditions, the maximum peptide mass concentration of 347.461 mg/g was achieved when using papain (PAP) at a 2.53% enzyme mass fraction, flavorzyme (FLA) at 4%, and bromelain (BRO) at 4%. This optimal enzymatic digestion process was repeated in three separate trials, with an average peptide mass concentration of 336.78 mg/g observed, which closely approximated the predicted value based on the regression model. These results validate the accuracy and reliability of the optimized parameters. The low deviation between the experimental and predicted values further supports the robustness of the regression model. Consequently, this model can be reliably employed to optimize the enzymatic hydrolysis process of chicken bones, offering a valuable tool for maximizing short peptide yields while minimizing the experimental variability.

### 3.3. Results of Peptide Molecular Weight Distribution Analysis

**Figure 3:**
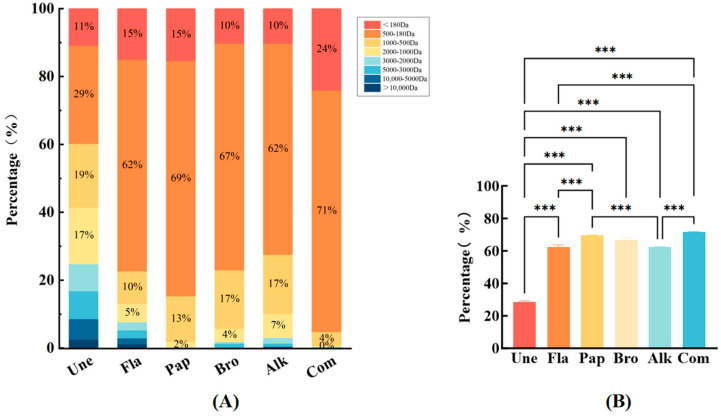
Molecular weight distributions. (**A**) Molecular weight distribution of the peptides after different enzymes; (**B**) comparison of the peptide content at 180–500 Da (*** *p* < 0.001).

Small molecule peptides, typically characterized by molecular weights ranging from approximately 180 to 480 Da and consisting of 2–10 amino acid chains, offer significant nutritional value due to their ease of absorption through the digestive tract (Figure 3A). These peptides are not only highly bioavailable, but also play a crucial role in enhancing the flavor profile of enzymatically digested products. The nutritional and flavor properties of enzyme digests are directly influenced by the specific small molecular peptides present within the hydrolysate [29]. Notably, low molecular weight peptides (LMWPs), which typically have molecular weights of less than 1 kDa, serve as important flavor precursors during the Maillard reaction. When subjected to heat, they contribute to the formation of volatile organic compounds (VOCs), which are critical for the development of distinct and desirable flavors [30]. Moreover, it has been observed that chicken broths containing peptide fractions smaller than 1 kDa exhibit enhanced freshness, and their overall flavor profile is more balanced and harmonious compared with broths with higher molecular weight peptides. Specifically, the 1–3 kDa peptide fractions from the enzymatic hydrolysis of chicken bones were found to significantly improve the perception of fresh flavor [31].

As illustrated in Figure 3B, under the optimal enzymatic digestion conditions, 95% of the peptides obtained from hydrolyzed chicken bones using a composite enzyme (COM) was smaller than 500 Da, a result that surpassed the peptide profiles obtained from single enzymes or non-enzymatic methods. The molecular weights of peptides derived from chicken by-product hydrolysates in other studies primarily fall within the 244 Da to 1.3 kDa range. These findings further underscore the influence of enzyme selection on the molecular weight distribution of chicken bone hydrolysates, suggesting that composite enzyme systems lead to higher yields of low molecular weight peptides [19]. This indicates that the composite enzyme hydrolysis of chicken bones can be an effective method for obtaining high concentrations of small molecular peptides, which are not only nutritionally beneficial, but also contribute to an enhancement in the product’s flavor profile.

### 3.4. Free Amino Acid Composition Analysis Results

**Figure 4:**
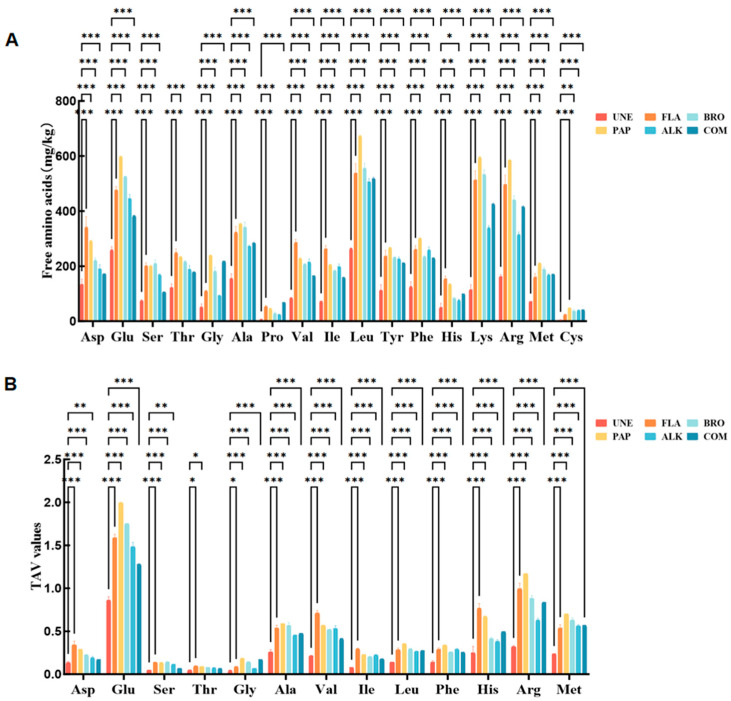
FAA content of the enzymatic hydrolysate products. (**A**) Individual free amino acid content of the unenzymatized chicken bones (UNE) and different enzymes; (**B**) TAV values of individual free amino acids of UNE and different enzymes (* *p* < 0.05, ** *p* < 0.01, *** *p* < 0.001).

During the degradation of proteins, these macromolecules are gradually broken down into smaller peptides, free amino acids (FAAs), and other organic compounds through enzymatic processes [32]. The wide range of amino acids released during this breakdown is crucial in contributing to the characteristic fresh, savory, and umami flavors commonly associated with meat-based products [33]. In this study, seventeen distinct amino acids were identified across six different enzyme-treated chicken samples (Figure 4). These amino acids were classified based on their specific taste attributes, which included the fresh, sweet, bitter, and tasteless profiles, according to the established flavor characteristic classifications [34].

As presented in Table 4, the total concentration of free amino acids in the unenzymatized chicken by-product group (UNE) was found to be significantly lower than that of the enzymatically treated groups, with values less than half of those observed in the other enzyme-treated samples. Free amino acids, as key precursors for various odor compounds, are pivotal in determining the flavor profile of enzymatic digests. Among all of the treatments, the highest concentration of free amino acids was found in the enzymatic products derived from the composite enzyme (COM) treatment, surpassing that of both the single and compound enzyme treatments. Notably, COM contained substantial quantities of flavor-enhancing amino acids, such as glutamic acid (Glu, 598.92 mg/g) and glycine (Gly, 240.24 mg/g), which were significantly higher than those in the hydrolysates obtained from other enzymes used for chicken bones [35]. Additionally, COM exhibited slightly higher concentrations of bitter amino acids, including arginine (Arg, 586.31 mg/g) and lysine (Lys, 594.81 mg/g), when compared to other monoenzymes. Amino acids, through biochemical transformations, can be converted into volatile compounds that impart distinct odors, thus contributing to the overall flavor experience. The taste activity values (TAVs) were employed as a standardized measure to assess the relative contribution of specific amino acids to the overall flavor profile. As shown in Table 4, the TAVs of glutamic acid (Glu) as well as other amino acids, such as aspartic acid (Asp), alanine (Ala), histidine (His), and arginine (Arg) in COM, were greater than 1, indicating a strong contribution to the flavor. Further research suggests that prolonged enzymatic digestion can reduce the intensity of bitterness, enhancing the overall sensory experience. After enzymatic digestion, the compound enzyme (COM) exhibited the most significant contribution to the development of fresh and sweet flavor amino acids, surpassing all of the other enzymatic treatments. This highlights the important role of COM in enhancing both the flavor and quality of the hydrolyzed chicken bone product, which is essential for optimizing taste and improving the sensory quality in food processing [36,37].

### 3.5. Results of Volatile Flavor Substance Analysis

Volatile flavor compounds, which contribute to odors, play a significant role in stimulating appetite and can indirectly influence food intake and digestion. In chicken meat, the predominant flavor compounds are water-soluble low molecular weight substances and lipids, along with various volatile compounds such as aldehydes, ketones, alcohols, acids, esters, hydrocarbons, furans, and nitrogen- and sulfur-containing compounds [38]. The formation of these flavor components is primarily driven by key synthetic pathways including the Maillard reaction, Strecker degradation, lipid oxidation, lipid–Maillard interactions, and thiamine degradation. To identify and quantify the volatile flavor compounds, headspace solid-phase microextraction gas chromatography-mass spectrometry (HS-SPME-GC-MS) was utilized, as summarized in Table 5. The total number of volatile flavor compounds in both the enzyme-digested and non-enzymatically treated chicken meat was analyzed using HS-SPME-GC-MS. A total of 55 compounds that exhibited statistically significant differences between the two groups (*p* ≤ 0.05, fold change (FC) ≤ 0.05 or ≥2), were identified. These compounds included three esters, eight alcohols, twenty-four aldehydes and ketones, and three sulfur ethers. The results indicate that there was no significant difference in the volatile flavor profile between the composite enzyme (COM) treated and unenzymatized chicken bones (UNE).

Aldehydes are primarily formed through the oxidation of fats and represent one of the key contributors to the flavor of meat [25]. The predominant aldehyde compounds identified in the study included trans-2-heptanal, E-2-octenal, benzaldehyde, hexanal, and nonanal (Table 5). Aldehydes in foods can impart fatty, grassy, and citrus-like flavors [39]. Alcohols, primarily derived from unsaturated fatty acids, were also prominent, with 1-octen-3-ol and hexanol standing out as key compounds that contribute mushroom-like flavors [40]. The acid content across all sample groups was relatively low. Notably, in comparison to the control group, three novel volatile compounds—propylnonanolactone, phenylmethyl isothiocyanate, and 2-ethylhexanol—were detected in the enzymatically digested chicken meat, with their concentrations showing a significant increase after enzymatic digestion.

Heterocyclic compounds, which contribute to a soybean-like flavor, are primarily generated via the Maillard reaction and the degradation of amino acids. Although their overall concentration was low, these compounds play a significant role in enhancing the flavor profile. Additionally, 2-pentylfuran was identified as imparting floral and fruity aromas [41]. Alkanes, due to their higher detection thresholds, contribute minimally to flavor. Overall, the results demonstrated a significant increase in both the variety and concentration of volatile compounds following enzymatic hydrolysis, resulting in a more pleasant and complex flavor profile.

## 4. Discussion

Enzymatic digestion was performed in this study to enhance the flavor and nutritional properties of raw chicken bones. Based on the univariate analysis, the optimum enzymatic digestion temperature was determined to be around 55 °C. The enzyme mass fraction and reaction time had the greatest influence on the enzymatic process. Proteases BRO, PAP, FLA and ALK with a high hydrolysis rate and low peptide mass concentration were selected for compounding in the range of 0–4% for durations of 1.5 h, 3 h, and 4.5 h, respectively. Optimization was carried out using response surface methodology to determine the ideal conditions for enzymatic hydrolysis: 55 °C for 1.5 h and enzyme mass fractions of 2.53%, 4%, and 4% for PAP, FLA, and BRO, respectively. Under these optimal conditions, the distribution of peptide molecular weights under these optimal conditions indicated that 95% of the peptides extracted from the enzyme complex were less than 500 Da, resulting in a high yield of low molecular weight peptides. Flavor compounds produced during enzymatic digestion, especially fresh and sweet amino acids, included Glu (598.92 mg/g) and Gly (240.24 mg/g). In terms of the taste activity values, the composite enzyme group showed that Asp, Ala, His, and Arg all exceeded 1, indicating their contribution to flavor. This underscores the significant role of the composite enzyme (COM) in enhancing both the flavor and overall quality of the hydrolyzed chicken bone product, which is crucial for optimizing taste and improving sensory attributes in food processing. In addition, the types and amounts of volatile compounds increased significantly after enzymatic digestion, especially the predominant aldehyde compounds identified including trans-2-heptanal, E-2-octenal, benzaldehyde, hexanal, and nonanal, presenting a meaty, fatty flavor, thus enhancing the flavor of canned meat, meat sausages, and pet foods including dog food, cat food, and canned food. The acid content was relatively low across all sample groups, indicating the absence of an acidic flavor. The acid content was relatively low across all sample groups and three new volatile compounds, propyl nonyl lactone, phenylmethyl isothiocyanate, and 2-ethylhexanol, were detected in the enzyme-digested chicken bones, with their concentrations increasing significantly after enzymatic digestion.

Although this study did not validate the nutritional value of the enzymatic process in vitro, it provides a valuable framework for further research and suggests a new method for enhancing the value of chicken bones. In our study, plant proteases, animal proteases, alkaline proteases, neutral proteases, and flavor proteases were used to provide a better research framework and data comparison for future nutritional and flavor studies of enzymatically digested chicken bones.

## 5. Conclusions

This study demonstrated that the flavour and nutritional properties of raw chicken bones can be enhanced through enzymatic hydrolysis. Enzyme screening was conducted via univariate experiments, with enzymes evaluated based on their biological activity. Enzymes exhibiting high hydrolysis rates and low peptide mass concentrations (BRO, PAP, FLA, and ALK) were selected for further analysis. Response surface methodology was employed to optimise the enzyme complexes across three distinct reaction times, with enzyme concentrations ranging from 0–4% and reaction durations of 1.5, 3, and 4.5 h. The optimal enzymatic conditions were determined to be 55 °C for 1.5 h, with enzyme mass fractions of 2.53%, 4%, and 4% for PAP, FLA, and BRO, respectively. Under these optimized conditions, 95% of the extracted peptides exhibited molecular weights below 500 Da, resulting in a high yield of low molecular weight peptides. Enzymatic digestion significantly increased the concentration of flavour compounds, particularly amino acids such as glutamic acid and glycine, which contributed to a sweet and savoury taste profile. Aspartic acid, alanine, histidine, and arginine displayed high taste activity values (TAVs), further enhancing the overall flavour. Moreover, the enzymatic process led to a marked increase in volatile compounds, predominantly aldehydes, including trans-2-heptanal, E-2-octenal, benzaldehyde, hexanal, and nonanal, which imparted rich meaty and fatty flavours, thus making the product suitable for use in meat products, sausages, and pet foods such as dog food, cat food, and canned food.

Although this study provides a method for enhancing the value of chicken bones, further research is required to validate the nutritional benefits of enzymatically digested chicken bones and to optimize the enzymatic conditions for broader applications in food processing and pet food production. This would facilitate the sustainable utilization of chicken bone resources and support the development of functional foods.

## Figures and Tables

**Table 1:** Factor and level design of the one-way test.

Factor	Process Group				
	1	2	3	4	5
Enzyme mass fraction E/S (%)	1	1.5	2	3	4
Enzymatic hydrolysis temperature/°C (T)	45	50	55	60	65
Enzymatic hydrolysis time/h (t)	1	2	3	4	5

**Table 2:** Independent variables used in response surface design and their levels.

Factor	Level		
	−1	0	1
*Alkaline protease* amount (%)	0	2	4
*Bromelain* amount (%)	0	2	4
*Papain* amount (%)	0	2	4
*Flavorzyme* amount (%)	0	2	4

**Table 3:** Response surface central composite design and peptide content.

Run	X1/Alkaline Protease Amount (%)	X2/Bromelain Amount (%)	X3/Papain Amount (%)	X4/Flavorzyme Amount (%)	1.5 h	3 h	4.5 h
1	0	0	2	2	189.11	190.22	180.89
2	4	2	2	4	199.22	227.78	195.11
3	2	2	4	0	154.67	284.89	147.11
4	4	0	2	2	107.11	145.00	136.44
5	2	2	2	2	258.22	253.11	295.56
6	2	4	4	2	189.56	134.67	191.56
7	0	2	2	4	211.11	254.67	244.89
8	0	2	2	0	137.33	127.11	135.56
9	0	2	0	2	101.78	134.22	191.56
10	4	4	2	2	231.56	271.56	119.56
11	2	2	2	2	260.13	253.78	306.10
12	2	4	0	2	148.89	149.78	172.89
13	2	2	0	0	256.44	228.00	298.22
14	4	2	2	0	254.10	267.11	303.56
15	2	0	2	0	269.78	256.44	271.56
16	2	2	2	2	255.30	251.20	298.40
17	0	2	4	2	403.11	205.78	78.67
18	4	2	4	2	240.44	225.33	168.44
19	2	2	2	2	254.30	254.60	295.56
20	2	2	2	2	255.60	258.10	297.20
21	2	2	0	4	106.78	167.56	302.67
22	2	4	2	4	211.89	218.67	304.44
23	4	2	0	2	117.78	202.22	230.67
24	2	0	0	2	113.78	180.00	140.89
25	2	0	4	2	190.67	213.78	153.33
26	2	0	2	4	105.33	222.67	107.11
27	2	2	4	4	225.33	224.44	177.33
28	0	4	2	2	193.33	158.67	198.67
29	2	4	2	0	161.33	189.78	161.33

**Table 4:** Free amino acid content and TAV values of different enzymes.

Free Amino Acid (mg/kg)		UNE	FLA	PAP	BRO	ALK	COM	Threshold (mg/k g)	TAVs (Wet Basis)					
									UNE	FLA	PAP	BRO	ALK	**COM**
Umani AA	Aspartic acid (*Asp*)	134.75 ± 18.47 ^e^	341.22 ± 38.91 ^a^	172.62 ± 0.86 ^d^	220.68 ± 9.29 ^c^	190.58 ± 14.74 ^cd^	291.51 ± 2.26 ^b^	1000	0.13 ± 0.02 ^e^	1.02 ± 0.04 ^a^	0.29 ± 0 ^b^	0.22 ± 0.01 ^c^	0.19 ± 0.01 ^cd^	0.17 ± 0 ^d^
	Glutamic acid (*Glu*)	258.85 ± 11.37 ^f^	477.66 ± 12.07 ^c^	382.13 ± 3.03 ^e^	525.26 ± 0.97 ^b^	445.31 ± 15.23 ^d^	598.92 ± 0.84 ^a^	300	0.86 ± 0.04 ^f^	4.78 ± 0.04 ^c^	2 ± 0 ^a^	1.75 ± 0 ^b^	1.48 ± 0.05 ^d^	1.27 ± 0.01 ^e^
Sweet AA	Serine (*Ser*)	75.51 ± 3.09 ^d^	202.41 ± 10.03 ^a^	105.9 ± 1.37 ^c^	210.06 ± 13.26 ^a^	167.94 ± 4.72 ^b^	200.72 ± 2.01 ^a^	1500	0.05 ± 0 ^d^	0.4 ± 0.01 ^a^	0.13 ± 0 ^a^	0.14 ± 0.01 ^a^	0.11 ± 0 ^b^	0.07 ± 0 ^c^
	Threonine (*Thr*)	123.06 ± 13.29 ^d^	249.01 ± 13.44 ^a^	178.59 ± 1.68 ^c^	215.55 ± 4.46 ^b^	187.52 ± 15.09 ^c^	234.1 ± 0.05 ^a^	2600	0.05 ± 0.01 ^d^	0.29 ± 0.01 ^a^	0.09 ± 0 ^a^	0.08 ± 0 ^b^	0.07 ± 0.01 ^bc^	0.07 ± 0 ^c^
	Glycine (*Gly)*	52.02 ± 10.72 ^f^	110.65 ± 0.16 ^d^	217.32 ± 2.04 ^b^	180.79 ± 15.44 ^c^	93.61 ± 1.88 ^e^	240.24 ± 0.58 ^a^	1300	0.04 ± 0.01 ^f^	0.26 ± 0 ^d^	0.18 ± 0 ^a^	0.14 ± 0.01 ^c^	0.07 ± 0 ^e^	0.17 ± 0 ^b^
	Alanine (*Ala*)	155.87 ± 16.81 ^d^	323.82 ± 19.98 ^b^	283.98 ± 2.76 ^c^	342.32 ± 17.98 ^ab^	272.86 ± 3.62 ^c^	354.23 ± 1 ^a^	600	0.26 ± 0.03 ^d^	1.62 ± 0.03 ^b^	0.59 ± 0 ^a^	0.57 ± 0.03 ^ab^	0.45 ± 0.01 ^c^	0.47 ± 0 ^c^
	Proline (*Pro*)	7.57 ± 0.08 ^f^	53.47 ± 3.71 ^b^	68.1 ± 0.94 ^a^	29.12 ± 4.24 ^d^	24.2 ± 2.13 ^e^	46.91 ± 0.52 ^c^	-	-	-	-	-	-	-
Bitter AA	Valine (*Val*)	84.72 ± 1.7 ^e^	284.81 ± 12.63 ^a^	164.54 ± 1.44 ^d^	207.78 ± 0.92 ^c^	214.07 ± 12.44 ^c^	227.89 ± 2.06 ^b^	400	0.21 ± 0 ^e^	2.14 ± 0.03 ^a^	0.57 ± 0.01 ^b^	0.52 ± 0 ^c^	0.54 ± 0.03 ^c^	0.41 ± 0 ^d^
	Isoleucine (*Ile*)	72.02 ± 1.04 ^e^	262.89 ± 12.14 ^a^	158.55 ± 1.89 ^d^	183.93 ± 1.89 ^c^	198.51 ± 9.18 ^b^	205.37 ± 1.82 ^b^	900	0.08 ± 0 ^e^	0.88 ± 0.01 ^a^	0.23 ± 0 ^b^	0.2 ± 0 ^c^	0.22 ± 0.01 ^b^	0.18 ± 0 ^d^
	Leucine (*Leu*)	264.03 ± 2.19 ^d^	538.26 ± 34.69 ^bc^	518.46 ± 4.85 ^c^	556.49 ± 18.1 ^b^	507.3 ± 10.97 ^c^	673.2 ± 2.83 ^a^	1900	0.14 ± 0 ^d^	0.85 ± 0.02 ^bc^	0.35 ± 0 ^a^	0.29 ± 0.01 ^b^	0.27 ± 0.01 ^c^	0.27 ± 0 ^c^
	Tyrosine (*Tyr*)	113.2 ± 20.06 ^d^	236.76 ± 20.79 ^b^	212.06 ± 1.26 ^c^	232.85 ± 0.46 ^bc^	225.58 ± 8.05 ^bc^	267.71 ± 0.75 ^a^	-	-	-	-	-	-	-
	Phenylalanine (*Phe*)	125.08 ± 18.3 ^d^	260.53 ± 14.2 ^b^	228.59 ± 2.6 ^c^	234.94 ± 2.71 ^c^	259.78 ± 10.15 ^b^	301.97 ± 0.45 ^a^	900	0.14 ± 0.02 ^d^	0.87 ± 0.02 ^b^	0.34 ± 0 ^a^	0.26 ± 0 ^c^	0.29 ± 0.01 ^b^	0.25 ± 0 ^c^
	Histidine (*His*)	49.76 ± 15.23 ^e^	153.23 ± 11.13 ^a^	99.18 ± 0.73 ^c^	82.75 ± 3.33 ^d^	75.93 ± 3.69 ^d^	135.03 ± 0.88 ^b^	200	0.25 ± 0.08 ^e^	2.3 ± 0.06 ^a^	0.68 ± 0 ^b^	0.41 ± 0.02 ^d^	0.38 ± 0.02 ^d^	0.5 ± 0 ^c^
	Lysine (*Lys*)	115.07 ± 17.08 ^e^	513.25 ± 33.11 ^b^	425.39 ± 4.29 ^c^	532.44 ± 17.77 ^b^	339.4 ± 7.9 ^d^	594.81 ± 2.82 ^a^	-	-	-	-	-	-	-
	Arginine (*Arg*)	162.29 ± 7.18 ^e^	496.7 ± 33.74 ^b^	415.55 ± 3.82 ^c^	440.97 ± 15.21 ^c^	314.6 ± 9.86 ^d^	586.31 ± 0.18 ^a^	500	0.32 ± 0.01 ^e^	2.98 ± 0.07 ^b^	1.17 ± 0 ^a^	0.88 ± 0.03 ^c^	0.63 ± 0.02 ^d^	0.83 ± 0.01 ^c^
Tasteless AA	Methionine (*Met*)	71.72 ± 0.56 ^d^	160.52 ± 12.73 ^c^	170.45 ± 1.48 ^c^	187.99 ± 8.47 ^b^	168.86 ± 4.23 ^c^	210.85 ± 0.53 ^a^							
	Cysteine (*Cys*)	4.48 ± 1.25 ^d^	23.05 ± 3.32 ^c^	41.08 ± 0.1 ^b^	37.37 ± 4.39 ^b^	40.49 ± 0.89 ^b^	48.78 ± 0.68 ^a^							
Tasteful AA (DAA)		1793.8 ± 87.47 ^e^	4504.66 ± 220.27 ^b^	3630.98 ± 30.67 ^d^	4195.92 ± 68.28 ^c^	3517.2 ± 121.54 ^d^	4958.91 ± 8.8 ^a^							
Umani AA (UAA)		393.59 ± 22.12 ^f^	818.88 ± 29.28 ^b^	554.76 ± 3.85 **^e^**	745.94 ± 9.88 ^c^	635.89 ± 29.63 ^d^	890.43 ± 3.09 ^a^	UAA/DAA (%)	35.92	29.03	31.23	32.30	31.79	27.63
Sweet AA (SAA)		414.04 ± 35.98 ^e^	939.36 ± 34.47 ^b^	853.89 ± 6 **^c^**	977.84 ± 27.11 ^b^	746.14 ± 23.67 ^d^	1076.19 ± 2.83 ^a^	SAA/DAA (%)	26.00	27.76	29.65	29.73	25.43	30.77
Bitter AA (BAA)		986.17 ± 41.37 ^e^	2746.42 ± 160.63 ^b^	2222.33 ± 20.82 ^d^	2472.14 ± 52.28 ^c^	2135.18 ± 68.88 ^d^	2992.29 ± 9.09 ^a^	BAA/DAA (%)	41.16	50.09	45.53	42.16	44.04	46.63
Total AA (TFAA)		1870 ± 86.81 ^e^	4688.22 ± 234.8 ^b^	3842.51 ± 32.24 ^d^	4421.28 ± 79.35 ^c^	3726.56 ± 126.27 ^d^	5218.54 ± 8.71 ^a^							

Note: Different letters meant there was significant difference among groups (*p* < 0.05).

**Table 5:** The composition of the main volatile substances and relative odor activity in the chicken enzymatic hydrolysate.

Category	RT/min	RI	Name	Content (µg/g FW)	
				UNE	COM
	10.93	1404	(E)-2-Octenal	4.08	42.37
	12.50	1522	(E,E)-3,5-Octadien-2-one	0.70	2.77
	8.72	1255.3	3-Octanone	0.02	0.12
	10.99	1408.5	(E)-3-Octen-2-one	0.62	4.74
	12.61	1530.5	Benzaldehyde	0.89	29.38
	14.47	1683.1	4-Ethyl-benzaldehyde	0.07	1.33
	14.08	1649.6	Benzeneacetaldehyde	0.10	4.60
	18.77	2087	*cis*-4-Decenal	0.17	2.82
	7.60	1185.6	Heptanal	1.35	13.09
	5.79	1080.5	Hexanal	40.11	214.29
	10.78	1393.7	Nonanal	3.60	33.65
	9.25	1289.4	Octanal	1.33	15.58
	4.06	975.4	Pentanal	2.07	12.92
	12.71	1538.2	*trans*-2-Nonenal	1.01	12.64
Subtotal				67.01	515.12
Alkanes	11.14	1419.8	5,5-Dimethyl-1,3-heptadiene	0.45	3.75
Alkane	7.58	1141	1,3-Dimethyl-benzene	0.06	14.53
	16.91	1903.3	*cis*-4,5-Epoxy-(E)-2-decenal	0.06	5.26
	7.76	1195.1	*D*-Limonene	0.12	0.98
	15.27	1753.2	Naphthalene	0.06	0.39
	2.12	837.3	Octane	0.48	6.96
	10.27	1358.2	3-Methyl-tridecane	0.05	0.18
Subtotal				1.28	32.05
Carboxylic acids	18.63	2073	2-Heptenoic acid	0.01	0.13
	11.58	1452.2	Acetic acid	0.10	2.77
	21.92	2438.3	Benzoic acid	0.01	0.19
	17.36	1946.5	Heptanoic acid	0.05	1.22
	16.24	1840.5	Hexanoic acid	1.66	16.63
	27.85	3055.6	n-Hexadecanoic acid	0.03	0.33
	19.45	2159.2	Nonanoic acid	0.05	0.54
	18.43	2052.6	Octanoic acid	0.12	1.88
	15.05	1734.1	Pentanoic acid	0.03	0.60
	24.45	2706.3	Tetradecanoic acid	0.02	0.29
Subtotal				2.09	24.57
Thioethers	17.27	1937.8	Benzyl nitrile	0.04	52.34
Sulfur (chemistry)	13.78	1624.7	2-(2-Propenyl)-furan	0.04	0.37
	8.33	1231.2	2-Pentyl-furan	0.39	18.17
Subtotal				0.47	70.88
Total				76.42	766.73

## Data Availability

The raw data supporting the conclusions of this article will be made available by the authors on request.

## Abbreviations

The following abbreviations are used in this manuscript:

UNE	Unenzymatized chicken bones
ALK	*Alkaline protease*
NEU	*Neutral Protease*
PAP	*Papain*
BRO	*Bromelain*
TRY	*Trypsin*
LEU	*Leucine Transaminase*
FLA	*Flavorzyme*
COM	Composite enzyme
DH	Hydrolysis degree
TCA	Trichloroacetic acid
FAA	Free amino acid
IS	Internal standard
RT	Retention time
RI	Retention index

## Appendix A

**Table A1:** Information on proteases used in this paper.

Protease Type	Protease Type	Site of Enzyme Action	Source
*Endonuclease*	*Alkaline protease*	Mainly catalyzes amide bonds formed by the carboxyl groups of hydrophobic amino acids, *Ala*, *Val*, *Gly*, *Leu*, *Ile*, *Phe*, *Trp*, *Met*, and *Pro.*	*Bacillus licheniformis*
*Endonuclease*	*Neutral Protease*	Peptide bonds containing aromatic amino acid residues such as *Tyr*, *Try*, and *Phe.*	*Bacillus amyloliquefaciens*
*Endonuclease*	*Papain*	*Papain* has a wide range of specificities and cleaves the peptide bonds of basic amino acids, *Leu* or *Gly*. It also hydrolyzes esters and amides.	*Papaya fruit*
*Endonuclease*	*Bromelain*	*Thioglycoproteinase*, no specific site of action.	*Jackfruit*
*Endonuclease*	*Trypsin*	C-terminal peptides of *Lys* and *Arg* residues are cleaved. Hydrolysis of the reaction slows down if the acidic residue is located on either side of the cleavage site and stops if the *Pro* residue is located on the carboxyl side of the cleavage site. *Trypsin* also cleaves the ester and amide bonds of the synthetic derivatives of amino acids.	*Pig pancreas*
*Exonuclease*	*Leucine Transaminase*	Hydrolysis of peptide chains formed at the N-terminal end of peptide chains from *Leu* and other amino acids, catalyzing the transfer of the α-amino group of leucine from the N-terminal end of the peptide chain to γ-glutamic acid.	*Bacillus subtilis* (bacillus subtilis)
A mixture of *Endonucleases* and *Exonucleases*	*Flavorzyme*	Hydrolysis of hydrophobic amino acids at the ends of short peptides.	*Aspergillus oryzae*
